# Direct ceramic coating of calcium phosphate doped with strontium via reactive growing integration layer method on α-Ti alloy

**DOI:** 10.1038/s41598-020-67332-8

**Published:** 2020-06-30

**Authors:** Chi Huang Huang, Masahiro Yoshimura

**Affiliations:** 1 Genomics Research Center, Academia Sinica, Taipei, Taiwan; 2 Hierarchical Green-Energy Materials (Hi-GEM) Research Center, Department of Material Science and Engineering, National Cheng Kung University, No. 1, University Road, Tainan, Taiwan

**Keywords:** Biomedical engineering, Biomedical materials

## Abstract

A strontium (Sr)-doped hydroxyapatite-like coating was deposited on α-Ti alloy via the growing integration layer (GIL) method at various applied voltages. We added 0.03 M strontium hydroxide (Sr(OH)_2_·8H_2_O) to a solution containing calcium acetate and sodium dihydrogen phosphate to produce Sr-doped hydroxyapatite (Sr-HA) coatings. The scanning electron microscope (SEM) images of these coatings showed that all various features, such as average pore size, coating thickness, micro-hardness, and roughness, were similar to those of HA. As the voltage increased from 250 to 300 V, the amount of micro cracks decreased, and there were eliminated at 350 V. The SEM images also showed that the Sr-HA coatings were closely integrated with the alloy: without any gaps between the oxide layers and the alloy. In addition, energy-dispersive X-ray spectroscopy verified the Sr integration from the bottom up. X-ray diffraction patterns confirmed Sr-HA formation instead of calcium phosphate, even at the lowest voltage of 250 V. The value of *E*_corr_ increased by 6.6% after raising the voltage from 250 to 350 V. The electrochemical impedance spectroscopy analysis confirmed that the adequate corrosion resistance of Sr-HA coatings, especially at the highest voltage of 350 V. In addition, the GIL treatment increased the layer resistance measured by R_p_/R_c._ Optimally, the GIL method used the highest voltage of 350 V to produce higher quality of Sr-HA-rich coatings.

## Introduction

Due to their favorable mechanical properties, titanium (Ti) alloys are considered excellent implant alloys^1,2^. Many kinds of Ti alloys were designed for applications in dental treatment and clinical surgery. Ti–Cu alloys have valuable mechanical properties like hardness and yield strength, and chemical properties, such as corrosion resistance and antibacterial effect. It is common belief that Ti–Cu alloy can improve by increasing the Cu contents^3^. The higher Cu content and fine Ti_2_Cu phase compounds increase the performance resulting in a stronger antibacterial ability and higher strength. In addition, the Cu ions do not lead to cell cytotoxicity. The alloys particularly exhibit very good cell biocompatibility and do not influence cell proliferation or differentiation even if the Cu ion content is increased to 25 wt%^4–6^. However, such alloys still need to be coated with a layer of biocompatible protective bioactive ceramics. Calcium phosphates are essential for such creamics. They improve corrosion protection of mechanical property. In addition, they enhance bone regeneration at the beginning apposition stage, and also regeneration of hard tissues in tissue engineering^7–10^. Among various calcium phosphates, HA is a major component of human bones and teeth^11,12^. During the past two decades, various surface treatments and deposition techniques for the HA coating of the Ti alloys have been studied. These techniques include plasma sprayed deposition, thermal spraying, ion beam assisted deposition (IBAD), sputtering, micro-arc oxidation (MAO), hot isostatic pressing, dip coating, electrophoretic deposition (EPD), and sol–gel coating^13–18^.

Recently, we proposed a novel coating method called growing integration layer (GIL)^19–21^ for the direct formation of coating layers on the Ti alloys via electrochemical reactions in solution at low temperature. The obvious difference between GIL strategy and MAO technology is the latter one to get oxide layer on the top of the sample surfaces. They are deposition of oxide layers by the top-on-top way. However, the GIL strategy is a concept to make integrated layers from bottom-to-top on a bulk material to eliminate the component (Cu) and form oxide layers composed TiO_2_, CaTiO_3_, and hydroxyapatite even including Sr. The result is a porous, uniform, and passive oxide coating on alloy surfaces with complex geometry. Due to its biocompatibility, GIL method is particularly suitable for dental (root) implants. As presented in our previous papers, the GIL process eliminates a major problem apparent for previous integration layer methods: stress formation and accumulation. Traditionally: (1) Layers are deposited one-by-one to integrate at relatively high temperature (300–500 °C or even 1,000 °C); (2) the interfaces accumulate stress during deposition and (annealing) cooling; (3) the stress accumulation may cause cracking, peeling, and deforming of the layers. The advantage of the GIL method is that an active element (A) can be added to the matrix component(s) (M) of the substrate alloy(s). During coating, appropriate reactions (oxidation, nitridation, carbonization, etc.) occur to yield coating layers like: AO, AN, and AC on the substrates. The coating layer grows from the inside of the alloy substrate to cover the surface of the substrate. GIL can be performed at low temperature, even in solution via electrochemistry, sono-chemistry, mechanochemistry, etc. Thus GIL coatings accumulate less stress, and adhere well. The process of GIL is non-destructive, taking place under relatively mild conditions. It can be repeated if another active component (A′) should be added to the body alloy. Therefore, GIL forms integrated layers from the bottom up, hence called “growing integration layer” method. If necessary, another coating layer can be produced on the substrate body by using another solution and interface reaction. Those secondary methods are can be called "reactive GIL"^21^, to distinguish from the basic GIL methods^19,20^. General ceramic coating technologies (e.g., electrodeposition, sol–gel, sputtering) may need multi-steps within heating, complex energetic deposition equipment, vacuum chamber system, or energy resources, etc. have been required in vacuum deposition.

Instead of only using Ca and P ions to synthesize a HA coating, recent surface treatments of Ti-alloy implants employed Zinc (Zn), Gallium (Ga), Magnesium (Mg), Selenium (Se), and Sr to improve the initial healing and promote faster healing times^22–24^. Sr occurs in small quantities in normal human bones, and it is believed to promote bone cell differentiation, inhibit bone resorption, reduce the bone loss rate, and it is used to treat osteoporosis^25–28^. However, they were reported that high doses of Sr cause bone abnormalities^29,30^.

For investigating the properties and electrochemical characteristics of the presence of Sr added to HA ceramic during the GIL method, we tried to produce Sr-HA coatings directly on a Ti_7_Cu_5_Sn alloy substrate at low temperature and atmospheric pressure. This paper also demonstrates that the micro-structure, composition, and corrosion resistance can be controlled through the voltage applied to grow Sr-HA coatings on the surface of an α-Ti alloy in Ca- and P-ion rich electrolyte.

## Materials and methods

The α-Ti alloy substrates were produced via arc-melting using α-Ti (88.0 wt%), Cu (7.0 wt%), and Sn (5.0 wt%). Next, the substrates were shaped into the specimens by wire electrical discharge machining (wire-EDM) cutting for the dimensions of 2 mm in length, 2 mm in width, and 20 mm in height. The specimens were polished with SiC abrasive papers (#100, #600, #1,000, #1,600, and #2,000 grit, in sequence). Then, the substrates were immersed in acetone, ethanol, and distilled water, and ultrasonically cleaned to remove contaminants and thin oxidation. For generating Sr-HA coatings on the substrates by the GIL method, 0.03 M (7.97 g/L) strontium hydroxide (Sr(OH)_2_·8H_2_O) was added into the electrolyte, containing 0.15 M (26.427 g/L) calcium acetate (CA) and 0.06 M (7.199 g/L) sodium dihydrogen phosphate (SDP). The mixed electrolyte was measured the pH value of 6.34. Then, the substrates were half way covered with Teflon tape keeping the exposed area only 0.84 cm^2^ and put in the above electrolyte to prepare five specimens with different applied voltages. The substrates were connected to a DC power supply as the anode. The cathode of the power supply was connected to a stainless steel vessel filled with the previously described electrolyte. The temperature of the electrolyte was controlled under 20 °C by a water cooling system to prevent too fast Sr-HA coating. The substrates were treated at various applied voltages (250 V, 300 V, and 350 V) with a current density of 3 A/cm^2^. The growing of Sr-HA coatings on the sample surfaces lasted 30 min.

The cross-section images, thickness, surface morphology, and composition of the samples were observed using a scanning electron microscope (SEM, S3000N, Hitachi, Japan) equipped with an energy-dispersive X-ray spectrometer (EDS). The phases, composition, crystal structure, and chemical composition of the coatings were determined by X-ray diffraction (XRD, D8 Advance, Bruker AXS, Germany) using Cu-Kα radiation with 40 kV and 30 mA in the 2θ range of 20°–80°. A scanning speed of 1°/min and a step size of 0.05° were used.

Hardness is one of the basic mechanical properties of material. To explore the GIL coatings' hardness, the Vickers micro-hardness was determined with a hardness test machine (HM 1 series-Mitutoyo, Japan) at different applied voltages. In order to avoid direct penetration of the GIL coatings, the instrument control parameters were maintained at 200 mg for load and at 10 s for dwell time. Altogether, 10 random locations were measured and averaged for hardness values.

Potentiodynamic polarization (PD) and EIS are commonly used for corrosion analysis. A PD test usually uses three electrodes, namely a working electrode, an Ag/AgCl reference electrode, and a platinum (Pt) assistant electrode. To evaluate the corrosion potential, PD analysis was carried out at a scan rate of 1 mV/s from − 1 to + 2 V using an electrochemical workstation (Jiehan 5000, Jiehan Technology Co., Taiwan). To determine the open-circuit potential, EIS measurements were conducted with an amplitude of 10 mV and a scanning frequency range from 10^5^ to 10^−2^ Hz. All electrodes were placed in 0.9 wt% NaCl solution at 37 ± 1 °C for the two electrochemical tests. During these tests, the exposed sample area of 0.84 cm^2^ was kept in contact with the 0.9 wt% NaCl solution. All EIS data were analyzed by curve fitting and using the circuit method from the Zview software package.

In addition, ions released from the GIL layers have been studied on selected specimens after the GIL treatment. The specimens were cleaned with alcohol and deionized water, then immersed in 7 ml of 0.9 wt% NaCl solution in a sealed glass bottle at room temperature (25 °C). The ion concentrations were analyzed after an immersion time of 7 days by an inductively coupled plasma-mass spectrometer (ICP-MS) (Agilent 7500ce, Agilent Technologies, Tokyo, Japan).

## Results and discussion

### Surface morphology and composition of Sr-HA coatings

**Figure 1:**
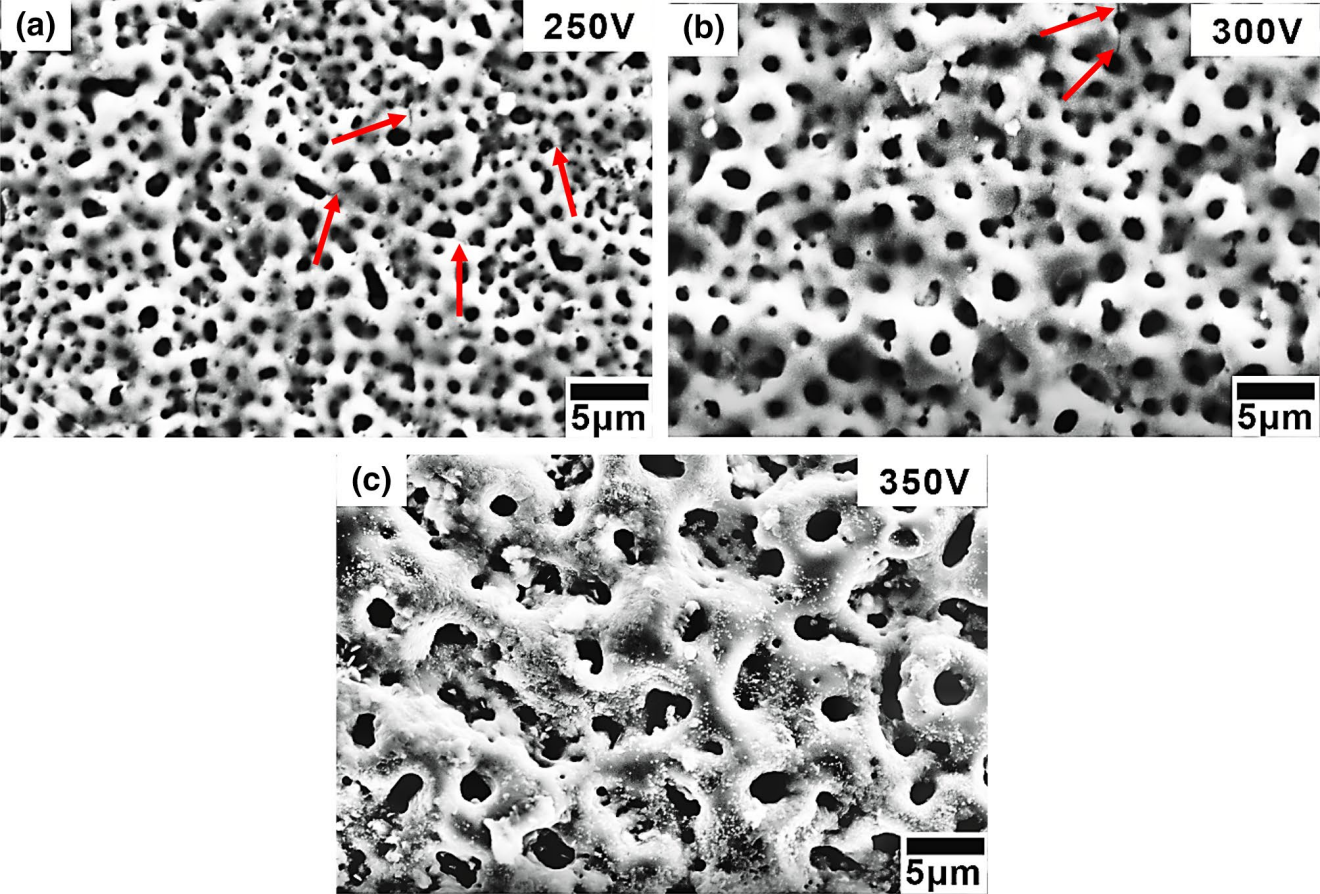
Surface morphology of GIL coatings obtained at 3 A/cm^2^ and applied voltages of (**a**) 250 V, (**b**) 300 V, and (**c**) 350 V.

The SEM pictures of surface morphology of the α-Ti alloy treated by GIL with various applied voltages in Sr-added electrolyte are shown in Fig. 1. The outside surface shows a porous network or sponge-like structure. Those might be beneficial for bone forming cell ingrowth, the adhesion of bacteria, and the adhesion of the alloy implant^31,32^. At lower voltages of 250 V and 300 V, the coatings had very less visible line cracks (shown in Fig. 1a, b). The crack occurrence is easy to find after surface modification process such as MAO or plasma sprayed method^16,26^. It is suspected that cracks caused by the slightly larger atomic radius of Sr ion occupied the original Ca ion position to induce lattice mismatch or stress formation during the oxidation at high temperature^16,33^. At higher voltage of 350 V, a great number of larger holes are well spread out on the surface, but there were no any cracks on the surface, as shown in Fig. 1c. It can proof that GIL method can successfully solve the cracks appearance and improve the coating surface performance to protect inside alloy.

**Figure 2:**
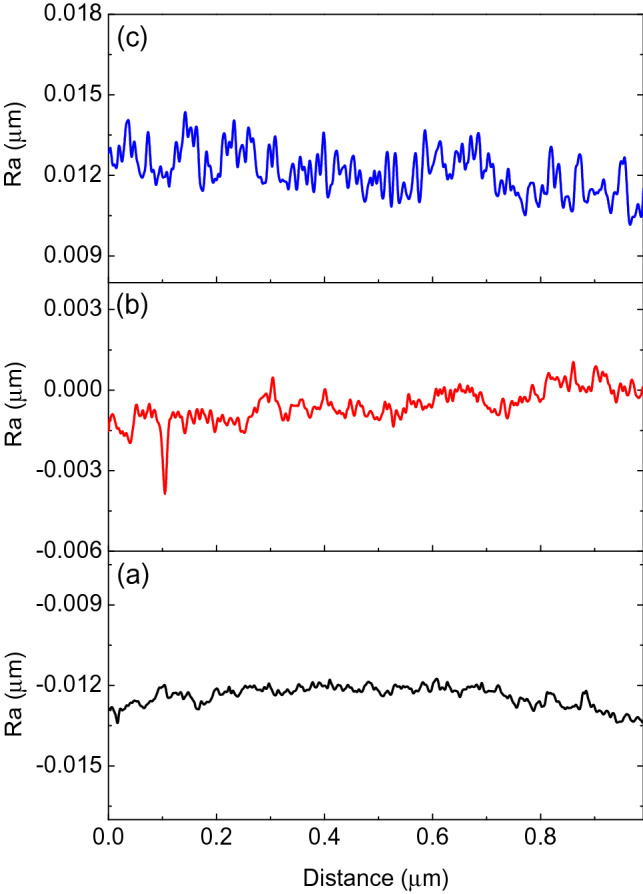
Surface roughness profiles of GIL coatings obtained at 3 A/cm^2^ and applied voltages of (**a**) 250 V, (**b**) 300 V, and (**c**) 350 V.

**Figure 3:**
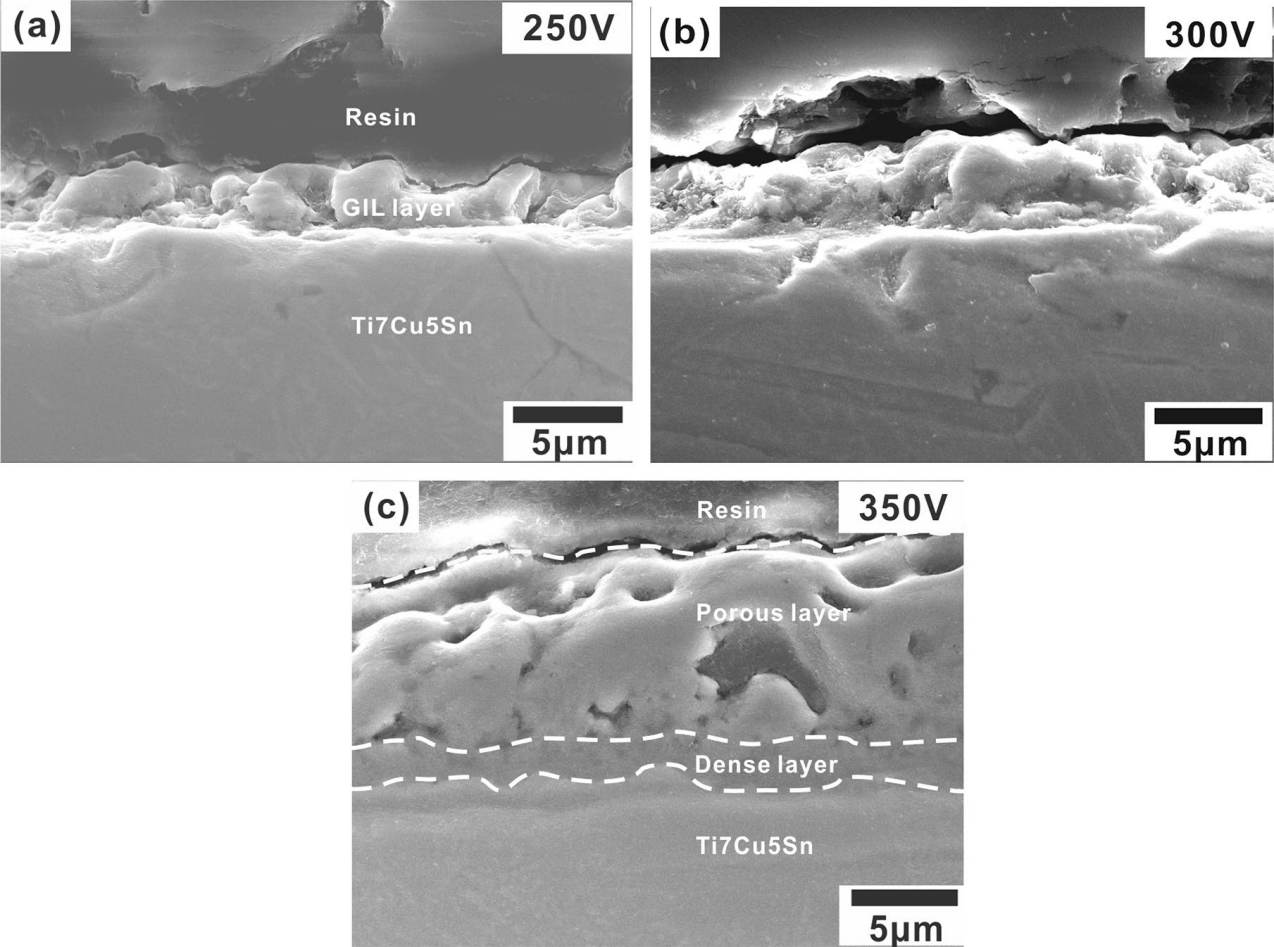
Cross-section images of GIL coatings obtained at 3 A/cm^2^ and applied voltages of (**a**) 250 V, (**b**) 300 V, and (**c**) 350 V.

The small holes were well isolated and homogeneously distributed on the surface, with an increasing average diameter of 0.9 to 2.2 μm from 250 to 350 V. The average thicknesses of GIL coatings also increased with the applied voltages. We measured 2.9, 4.2, and 10.9 μm, respectively. The hardness also increased with voltage, as shown in Table 1. The surface roughnesses (R_a_) of GIL coatings were determined by an alpha-step IQ surface profiler (KLA Tencor, KLA-Tencor Corporation, USA) and the highest one was at 350 V, as seen in Fig. 2. The results for R_a_ were 86.3 nm, 186.5 nm and 347.9 nm, see Table 1. By increasing the applied voltage, the average size, thickness, hardness, and roughness were all increased. Moreover, there was no significant coating separation or peeling between the GIL coatings and the α-Ti alloy substrate with the three applied voltages. Our GIL coatings showed good adhesion to the substrate, see Fig. 3. The cross section area of a typical sample treated at 350 V shows two distinct regions: a dense inside layer and a porous outside layer, see Fig. 3c.

**Table 1:** Average size, thickness, and hardness of GIL coatings.

Sample	Average size (μm)	Thickness (μm)	Hardness (Hv)	R_a_ (nm)
α-Ti alloy	na	na	431.4 ± 7.09	na
GIL250S	0.9 ± 0.3	2.9 ± 0.4	505.45 ± 23.85	86.3
GIL300S	1.6 ± 0.4	4.2 ± 0.4	532.53 ± 29.23	186.5
GIL350S	2.2 ± 0.7	10.9 ± 0.9	570.14 ± 40.56	347.9

### XRD patterns of GIL coatings

**Figure 4:**
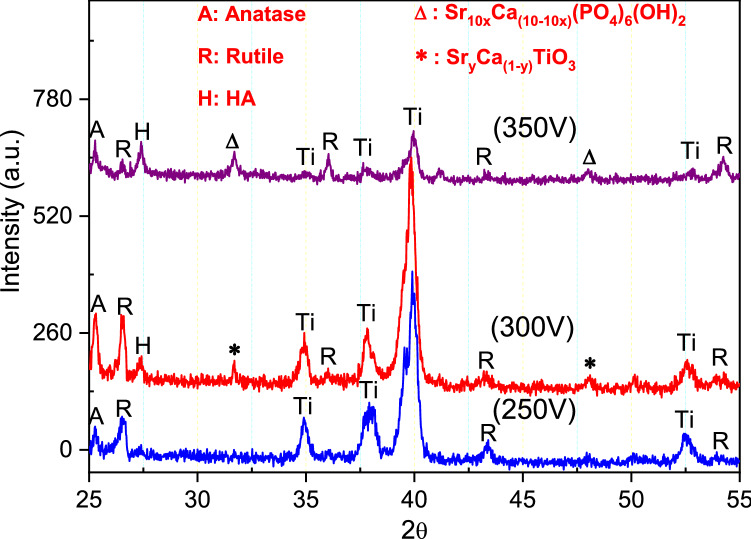
XRD patterns of GIL coatings obtained at various voltages.

Figure 4 displays the XRD patterns of GIL coatings on α-Ti alloy for various applied voltages with 30-min oxidation time, including Ti (JCPDS 44-1294 card), HA (JCPDS 09-432 card), anatase-TiO_2_ (JCPDS 21-1272 card), rutile-TiO_2_ (JCPDS 21-1276 card). The peaks of rutile, anatase, and Ti were found in the spectrum at 250 V, see the blue curve in Fig. 4. The peak of the spectrum for 250 V indicates that anatase and rutile were not well crystallized, as shown by the poor shape of the peak and the low value of full width at half the maximum. With 300 V, more peaks appeared in the spectrum, corresponding to anatase, rutile, HA, Ti, and Sr_y_Ca_(1−*y*)_TiO_3_. The magnitudes of the rutile, anatase, and HA peaks were respectively greater than those for 250 V, showing more crystallinity by the red curve in Fig. 4. The HA precursor of Sr_y_Ca_(1−*y*)_TiO_3_ already can been found. In addition, for 350 V, more well crystalline phase peaks have been detected and the peaks of anatase, rutile, HA, and Sr_10*x*_Ca_(10−10*x*)_(PO_4_)_6_(OH)_2_ represent the main phase in the coatings, as shown by the purple curve in Fig. 4. Especially, the GIL layers successful transform from Sr_y_Ca_(1−*y*)_TiO_3_ to Sr_10*x*_Ca_(10−10*x*)_(PO_4_)_6_(OH)_2_. Moreover, crystalline TiO_2_ phases and apatite-like phases of Sr, Ca, and P were also detected in all GIL coatings. In addition, a Sr–Ca–P–O amorphous phase might have been formed during GIL processing. Similar results were also obtained in previous studies. Han et al. found that the MAO coating had a mixture of rutile and anatase phases at high voltage of 380 V or 500 V^34,35^. When working with the reactive GIL method, the predominant phases changed from anatase and rutile to HA as the voltage increased from 300 to 350 V. Dicalcium phosphate dihydrate, a thermodynamically unstable and comparatively soluble material, is a superior precursor to generate HA^36^. Since temperature and energy increase with voltage during the GIL process, Sr, Ca, and P ions become more reactive to form Sr-HA at the surface of the α-Ti alloy. The amount of Sr-HA thus increased with voltage.

Ca^2+^, HPO_4_^2−^, CH_3_COO^−^, PO_4_^3−^ and OH^−^ derived from the dissociation of ionized CA, SDP, and H_2_O, respectively, were incorporated into the GIL coatings to form HA (Ca_10−*x*_(HPO4)_*x*_ (PO_4_)_6−*x*_(OH)_2−*x*_, 0 ≤ x ≤ 1, e. g., d**-**HA)^37^.

When voltage is applied, the following reactions occur at the α-Ti alloy surface:

3a $$ {\text{O}}_{2} + 2{\text{H}}_{2} {\text{O}} + 4{\text{e}}^{ - } \to 4{\text{OH}}^{ - } $$

3b $$ {\text{CH}}_{3} {\text{COO}}^{ - } + {\text{H}}_{2} {\text{O }} \leftrightarrow {\text{CH}}_{3} {\text{COOH}} + {\text{OH}}^{ - } $$

3c $$ 2{\text{H}}_{2} {\text{O}} + 2{\text{e}}^{ - } \to {\text{H}}_{2} + 2{\text{OH}}^{ - } $$

Reactions (3a–3c) result in a local jump of the pH value in the vicinity of the cathode, leading to the following chemical reactions:

3d $$ {\text{H}}_{2} {\text{PO}}_{4}^{ - } + {\text{OH}}^{ - } \to {\text{H}}_{2} {\text{O}} + {\text{HPO}}_{4}^{2 - } $$

3e $$ 2{\text{H}}_{2} {\text{PO}}_{4}^{ - } + 2{\text{e}}^{ - } \to 2{\text{HPO}}_{4}^{2 - } + {\text{H}}_{2} $$

3f $$ {\text{H}}_{2} {\text{PO}}_{4}^{ - } \leftrightarrow {\text{HPO}}_{4}^{2 - } + {\text{H}}^{ + } $$

Then, HPO_4_^2−^ combines with Ca^2+^ and Sr^2+^ to produce a Ca_1−*x*_Sr_*x*_HPO_4_·2H_2_O precipitate, deposited on the α-Ti alloy surface:

3g $$ x{\text{Sr}}^{2 + } + \left( {1 - x} \right){\text{Ca}}^{2 + } + {\text{HPO}}_{4}^{2 - } + 2{\text{H}}_{2} {\text{O}} \to {\text{Sr}}_{x} {\text{Ca}}_{(1 - x)} {\text{HPO}}_{4} \cdot 2{\text{H}}_{2} {\text{O}}\quad \left( {1 > {\text{x}} > 0} \right) $$

At the same time, OH^−^ in the vicinity of the cathode ignites the following reaction^38,39^:

3h $$ 10{\text{Sr}}_{x} {\text{Ca}}_{(1 - x)} {\text{HPO}}_{4} + 2{\text{OH}}^{ - } \to {\text{Sr}}_{10x} {\text{Ca}}_{(10 - 10x)} \left( {{\text{PO}}_{4} } \right)_{6} \left( {{\text{OH}}} \right)_{2} + 4{\text{PO}}_{4}^{3 - } + 10{\text{H}}^{ + } \quad \left( {1 > {\text{x}} > 0} \right) $$

The formation of Sr-doped Ca phases is the first evidence of this reactive GIL method.

### EDS analysis of Sr-HA coating

**Figure 5:**
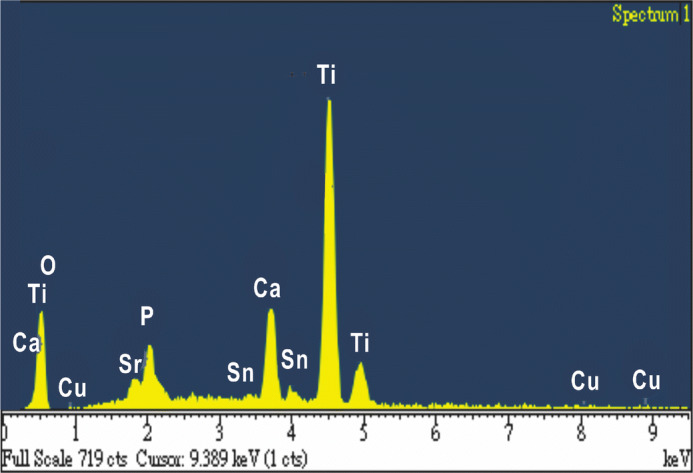
EDS spectrum of Sr-HA coatings obtained at applied voltage of 350 V and current of 3 A/cm^2^.

**Figure 6:**
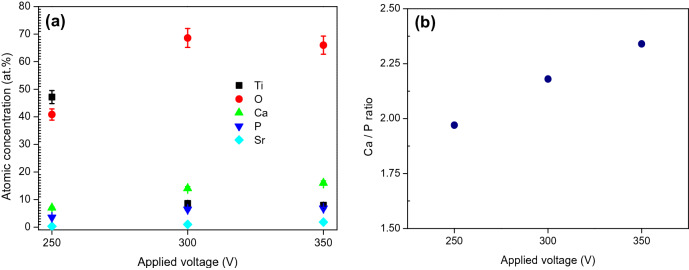
(**a**) Composition of GIL coatings obtained at various voltages and fixed current density of 3 A/cm^2^; (**b**) Ca/P ratio.

The composition of Sr-HA coatings on the surface of α-Ti alloy after GIL treatment was evaluated through EDS (Fig. 5). Large amounts of Ca, P, O, and Ti were found in all specimens using a current density of 3 A/cm^2^. Small amounts of Cu, Sn, and Sr were also detected in the coatings. The Cu and Sn came from the α-Ti alloy, while Sr came from the electrolyte. A thick layer of Sr-HA can hinder the Cu and Sn from the substrate. At 250 V, the total contents of Ti and O were 47.16% and 40.84%, respectively. This suggests that a lot of the TiO_2_ compounds were synthesized in the coatings, as shown in Fig. 6a. However, at 350 V, the Ca/P ratio reached 2.34, which is much larger than that found in human bone (1.67) (Fig. 6b and Table 2). At 350 V, the Ti content decreased to 7.94%, while the concentrations of O, Ca, P, Cu, Sn, and Sr all increased. Table 2 shows the ratios of (Ca + Sr)/P in the coatings, as 2.06%, 2.33%, and 2.6% for GIL250S, GIL300S, and GIL350S, respectively. Increasing the applied voltage to create stronger electric field enhanced the driving force for the reactions, increasing the content of Ca, P and Sr. It also proves that Sr ions were successfully added to the coated surface with GIL. Some studies report that precise control over the concentration of Ca and Sr in the coating is a key factor to enhance the cell cytocompatibility, osseointegration, and the bone healing effect of endosseous implants, because an excessively high concentration of Sr in the coating would be harmful to cell growth and would induce defective bone mineralization^40,41^. Therefore, EDS analysis results affected by voltage parameter can well support us to obtain the ideal Ca/P or (Ca + Sr)/P ratios of GIL coatings.

**Table 2:** Composition of Sr-HA coatings.

Sample	Element (at.%)								
	Ti	O	Ca	P	Cu	Sn	Sr	(Ca + Sr)/P	Ca/P
α-Ti alloy	85.12	na	na	na	12.75	2.13	na	na	na
GIL250S	47.16	40.84	6.98	3.54	0.35	0.83	0.30	2.06	1.97
GIL300S	8.59	68.61	14.02	6.44	0.40	0.95	0.99	2.33	2.18
GIL350S	7.94	65.99	16.00	6.85	0.42	0.98	1.82	2.60	2.34

**Figure 7:**
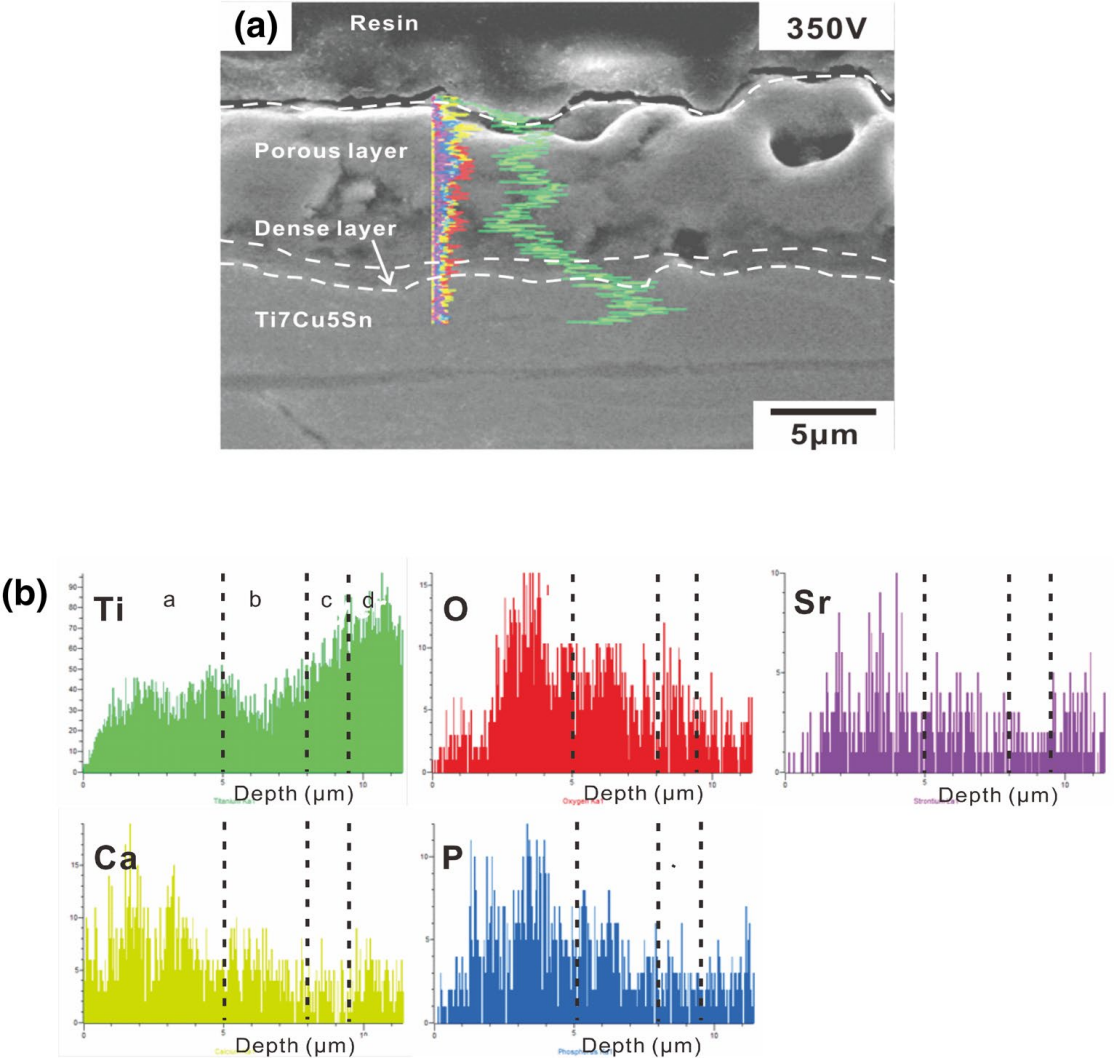
(**a**) Cross-section SEM image of Sr-HA; (**b**) EDS spectra of Sr-HA coating obtained at 350 V and 3 A/cm^2^.

A cross-sectional SEM image and the composition of Sr-HA coatings on α-Ti alloy are shown in Fig. 7. Many micro- to macro-holes appear in the outer oxide layer (porous layer). In addition, a dense layer and a thin oxide layer can be detected between the porous layer and the α-Ti alloy, as shown in Fig. 7a. There are no obvious discontinuities or gaps between the coating and the alloy substrate. This indicates that the coating is tightly integrated with the alloy substrate. The coating thickness is approximately 9.6–12.1 μm. The EDS spectra indicate that the GIL coating could roughly be divided into three parts, namely (1) the TiO_2_-rich compact inner layer (area b), (2) the middle layer, consisting of a mixture of porous TiO_2_ and HA compounds (area c), and (3) the porous outer layer consisting of HA compounds with Sr (area d) (Fig. 7b). The α-Ti alloy substrate is depicted by area a.

### Corrosion behavior of Sr-HA coatings by potentiodynamic (PD) polarization

**Figure 8:**
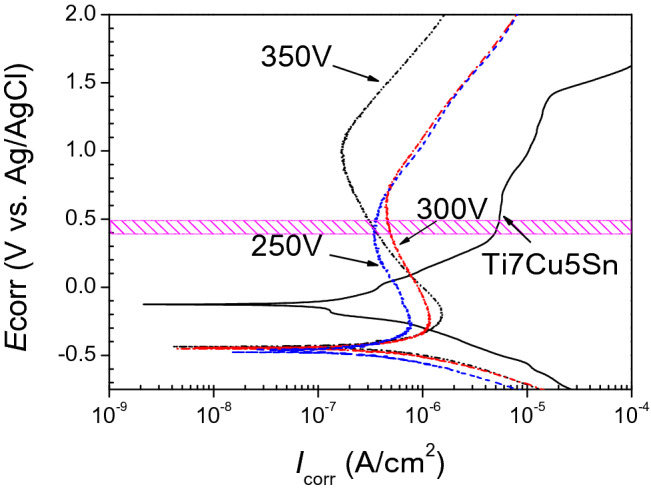
PD curves of bare α-Ti alloy substrate and substrate with Sr-doped GIL coating in 0.9 wt% NaCl solution.

PD tests were used to determine the corrosion rate and the fine internal structure of the coatings. The representative PD curves for untreated α-Ti alloys and for GIL coatings containing Sr (in 0.9 wt% NaCl solution at 37 ± 1 °C) are shown in Fig. 8. The related corrosion values are listed in Table 3. The corrosion potential (*E*_corr_) and the corrosion current density (*I*_corr_) were calculated from the curves using the Tafel extrapolation method. The *E*_corr_ value for the untreated alloy substrate is about − 127.7 mV with an associated *I*_corr_ = 167.7 nA/cm^2^ versus Ag/AgCl, which is a larger value for *E*_corr_ than those for Sr-HA coatings. As the applied voltage increased from 250 to 350 V, the values of *E*_corr_ for Sr-HA coatings shifted from − 466.5 to − 435.9 mV which increased by 6.6%. This means that Ti and its alloys produce a compact oxide layer on the alloy surface with higher corrosion resistance. Thus, the Sr-HA coating layers can prevent ion release/dissolution (including Cu) from the alloys, acting as effective corrosion barriers. Furthermore, the upper halves of the anodic polarization curves show an inflection at the second turning point, the so called breakdown potential (*E*_b_) for three GIL coatings. This phenomenon corresponds to the pitting corrosion of Ti alloys. By increasing the applied voltage from 250 to 350 V, the *E*_b_ values shifted from 434.5 to 1,025.8 mV, indicating the retardation of pitting corrosion. GIL layers at 350 V reached the maximal value for Δ*E* (= *E*_b_ − *E*_corr_) which reveals the low dissolution property of the substrate alloys when having the higher Δ*E*.

**Table 3:** Corrosion potential and current of bare α-Ti alloy substrate and substrate with Sr-HA coating.

Sample	*E*_corr_ (mV_Ag/AgCl_)	*I*_corr_ (nA/cm^2^)	*E*_b_ (mV_Ag/AgCl_)	Δ*E* (mV)
α-Ti alloy	− 127.7	167.7	1,415.7	1543.4
GIL250S	− 466.5	304.5	434.5	901.0
GIL300S	− 447.5	473.5	608.3	1,055.8
GIL350S	− 435.9	989.0	1,025.8	1,461.7

### Corrosion behavior of Sr-HA coatings by electrochemical impedance spectroscopy (EIS)

**Figure 9:**
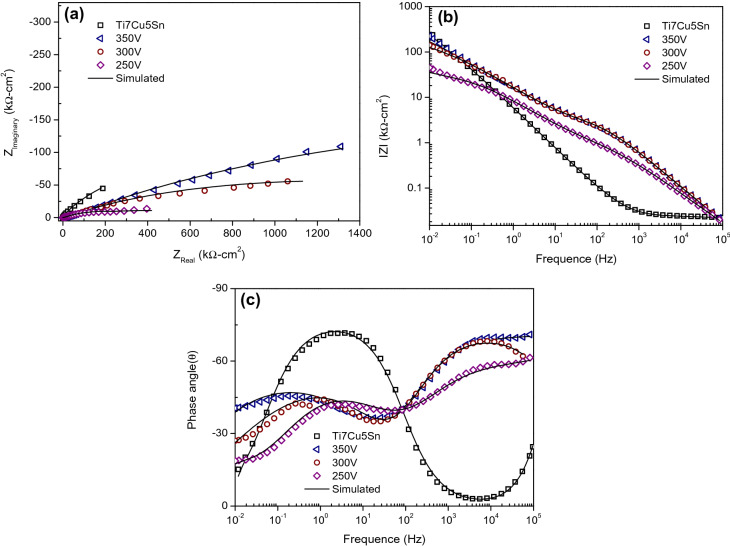
(**a**) Nyquist plot; (**b**) bode impedance plot; (**c**) bode phase angle plot of bare α-Ti alloy substrate and substrate with Sr-HA coatings at various voltages in 0.9 wt% NaCl solution.

**Figure 10:**
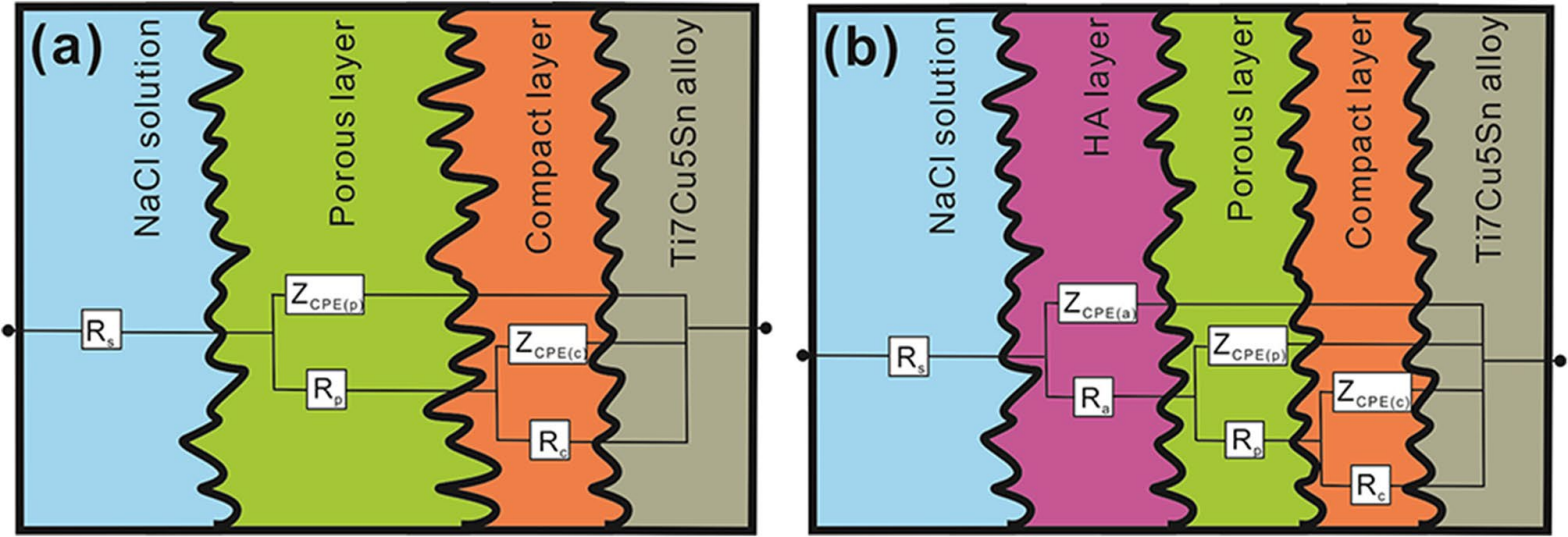
The detail structures and corresponding equivalent circuits used for fitting EIS spectra of (**a**) uncoated α-Ti alloy substrate; (**b**) substrate plus GIL layers.

EIS plots were obtained for the untreated alloy substrate and for Sr-HA coatings in 0.9 wt% NaCl solution at 37 ± 1 °C to characterize the corrosion behavior. In Fig. 9a, the Nyquist plots are impedance diagrams obtained for the untreated α-Ti alloy substrate and for the Sr-HA coated specimens. The untreated Ti has higher corrosion resistance than Sr-HA coatings (i.e., it has the largest semicircle radius). In Fig. 9c, the Bode plot of three valleys for all Sr-HA coatings indicate the existence of three time-constants in the experimental frequency range from 10^5^ to 10^−2^ Hz. Probably, the three-layer passive coating for Sr-HA coatings brings about the presence of two thick outer layers and one thin inner layer. Accordingly, the three constant phase elements: Z_CPE(a)_, Z_CPE(p)_, and Z_CPE(c)_ relate to the capacitances of the Sr-HA-rich layer, the calcium phosphate compounds layer, and the TiO_2_ oxide layer, respectively. The four resistance components, R_s_, R_a_, R_p_, and R_c_ relate to the solution's resistance, the porous outer layer's resistance (Sr-HA-rich), the porous middle layer's resistance (calcium phosphate compounds), and the dense inner layer's resistance (TiO_2_), respectively. The equivalent circuit models can be designed as shown in Fig. 10 according to the best fitted results for the α-Ti alloy substrate and for the Sr-HA coated specimens, respectively. The values of these parameters fitted by Zview software are listed in Table 4. For the α-Ti alloy, R_c_ is higher than R_p_ indicating that the inner barrier layer has higher corrosion resistance than the porous middle layer for all Sr-HA coated specimens.

**Table 4:** The EIS parameters of bare α-Ti alloy substrate and substrate with Sr-HA coating.

Sample	α-Ti alloy	GIL250S	GIL300S	GIL350S
R_S_ (Ω–cm^2^)	13.25 ± 2.32	14.29 ± 0.50	15.19 ± 0.37	14.75 ± 0.45
Z_CPE(a)_ (μF–cm^−2^)	–	1.45 ± 0.59	1.51 ± 0.15	1.58 ± 0.05
Z_CPE(p)_ (μF–cm^−2^)	4.90 ± 0.45	38.33 ± 0. 28	46.60 ± 0. 36	51.25 ± 0.41
Z_CPE(c)_ (μF–cm^−2^)	30.79 ± 0.49	108.62 ± 1.30	98.33 ± 2.91	89.75 ± 0.22
n_a_	–	0.66 ± 0.01	0.79 ± 0.01	0.78 ± 0.01
n_p_	0.47 ± 0.04	0.60 ± 0.01	0.76 ± 0.09	0.81 ± 0.01
n_c_	0.90 ± 0.01	0.86 ± 0.02	0.50 ± 0.02	0.57 ± 0.13
R_a_ (kΩ–cm^2^)	–	1.45 ± 0.10	3.50 ± 0.62	3.27 ± 0.13
R_p_ (kΩ–cm^2^)	1.34 ± 0.01	34.81 ± 0.78	37.70 ± 0.78	45.54 ± 11.20
R_c_ (kΩ–cm^2^)	805.35 ± 64.58	115.33 ± 5.25	261.11 ± 5.25	601.95 ± 14.13
χ^2^ (× 10^−3^)	2.51 ± 0.03	1.72 ± 0.13	1.30 ± 0.79	1.10 ± 0.09

From Ohm’s law, R_a_, R_p_, and R_c_ are proportional to pore depth and inverse proportional to coating porosity (i.e., porous area). Therefore, the R_c_ values for specimens at 300 V and 350 V are higher than that obtained at 250 V. This is due to the thicker compact inner layer. The same phenomenon occurs in the porous layers (R_p_) of GIL samples (34.81 and 45.54 kΩ–cm^2^ at 250 V and 350 V, respectively). These results show that the total thickness and resistance of GIL coatings are mostly contributed by the compact inner oxide layer. In addition, R_a_ is lower than R_p_ and R_c_ owing to the high porosity and the low conductivity of Sr-HA coatings. The constant phase elements Z_CPE(a)_, Z_CPE(p)_, and Z_CPE(c)_ can be used to fit the EIS data by varying parameter n between real numbers 0 and 1. Since surface heterogeneities are caused by the roughness of either the TiO_2_ (oxide) layer or the Sr-HA coating, Z_CPE(a)_, Z_CPE(p)_, and Z_CPE(c)_ should not stand for pure capacitors. Thus, n_a_ being close to 1 (350 V–3 A/cm^2^) would imply good resistance. The total impedance values (R_s_, R_a_, R_p_, and R_c_) are equivalent to the y-intercepts of the Bode impedance curves in Fig. 9b. Thus, higher intercept values mean larger coating resistance and better corrosion resistance. The maximum phase angles on the Bode plot for a Sr-HA coated specimen at 250 V, 300 V, and 350 V in the lower frequency region are − 43°, − 44°, and − 46°, while in the higher frequency region, they are − 60°, − 67°, and − 70°, respectively. According to the above results and discussion, we can conclude that the GIL treatment at high voltage can produce excellent corrosion resistance of Sr-HA ceramic to protect the inside alloy substrate.

### Ion release concentration from Sr-HA coatings

The concentration of ions released from Sr-HA coatings after immersion in NaCl solution was measured by inductively coupled plasma mass spectrometry, and the results are shown in Table 5. We found a trace amount of Ti, Cu, Sn and Sr ions in the solution after 7 days of immersion. The concentrations of Ti, Cu, and Sn ions were a few hundred times lower than those of Ca and P ions. This means that the thicker Sr-HA coating hinders ion movement and reduces their escape from the coatings. The increase of applied voltage from 250 to 350 V reduced the quantity of Ti, Cu, and Sn ions from 0.014 to 0.01 ppm, from 0.127 to 0.045 ppm, and from 0.208 to 0.163 ppm, respectively. Thus we can conclude that the reactive GIL method is very effective in the elimination of toxic Cu dissolution. On the other hand, the dissolution of Sr ions showed an obvious increase with voltage from 0.097 to 0.341 ppm, while Ca and P ion concentrations remained constant between voltage changes.

**Table 5:** ICP-MS results of Sr-HA coatings.

Sample	Element (ppm)					
	Ti	Cu	Sn	Ca	P	Sr
GIL250S	0.014	0.127	0.208	7.060	1.459	0.097
GIL300S	0.012	0.113	0.182	7.370	1.464	0.158
GIL350S	0.010	0.045	0.163	7.886	1.823	0.341

## Conclusions

In this study, we demonstrated that the reactive GIL method can imply produces Sr-HA coatings on α-Ti alloy substrate surfaces at room temperature. Using 0.15 M CA, 0.06 M SDP, and 0.03 M strontium hydroxide, we produced Ca/P-rich and Sr-HA-rich coatings on the surface of α-Ti alloy by applying three voltages of 250 V, 300 V and 350 V within the same current density of 3 A/cm^2^ for 30 min. The average pore size, thickness, micro hardness, and surface roughness of the coating at 350 V increased obviously compared to the similar values obtained at 250 V. As the voltage increased from 250 to 300 V, the amount of micro cracks decreased, and they got eliminated at 350 V. Hence, the applied voltage is the key parameter in the GIL process. The GIL treatment also effectively enhances the interface adhesion between ceramic coatings and metallic substrates, and decreases the thermal stress accumulation in the coating during processing. The PD test results showed the corrosion potential for α-Ti alloy as − 127.7 mV. We attribute this to the dense oxide film on its surface. The corrosion potential for Sr-HA coatings were − 435.9 mV, − 447.5 mV, and − 466.5 mV, for the different applied voltages. These are all lower than that of the α-Ti alloy since there are numerous calcium phosphates, HA, and Sr-HA compounds present in the coating. The value of *E*_corr_ increased by 6.6% from − 466.5 to − 435.9 mV after raising the voltage from 250 to 350 V. Since, the GIL process does not require vacuum or any other complicated equipment, it consumes little energy. The GIL technology can be used for not only biological engineering and biomedical science, but also in wider areas of science and engineering.

## References

[1] Tsao LC (2015). Effect of Sn addition on the corrosion behavior of Ti–7Cu–Sn cast alloys for biomedical applications. Mater. Sci. Eng. C.

[2] Cordeiro JM (2017). Development of binary and ternary titanium alloys for dental implants. Dent. Mater..

[3] Zhang E, Ren J, Li S, Yang L, Qin G (2016). Optimization of mechanical properties, biocorrosion properties and antibacterial properties of as-cast Ti–Cu alloys. Biomed. Mater..

[4] Liu J (2014). The antibacterial properties and biocompatibility of a Ti–Cu sintered alloy for biomedical application. Biomed. Mater..

[5] Zhang E, Zheng L, Liu J, Bai B, Liu C (2015). Influence of Cu content on the cell biocompatibility of Ti–Cu sintered alloys. Mater. Sci. Eng. C.

[6] Zhang E, Wang X, Chen M, Hou B (2016). Effect of the existing form of Cu element on the mechanical properties, bio-corrosion and antibacterial properties of Ti–Cu alloys for biomedical application. Mater. Sci. Eng. C.

[7] Mhaede M, Pastorek F, Hadzima B (2014). Influence of shot peening on corrosion properties of biocompatible magnesium alloy AZ31 coated by dicalcium phosphate dihydrate (DCPD). Mater. Sci. Eng. C.

[8] Imaizumi H, Sakurai M, Kashimoto O, Kikawa T, Suzuki O (2006). Comparative study on osteoconductivity by synthetic octacalcium phosphate and sintered hydroxyapatite in rabbit bone marrow. Calcif. Tissue Int..

[9] Bose S, Tarafder S (2012). Calcium phosphate ceramic systems in growth factor and drug delivery for bone tissue engineering: a review. Acta Biomater..

[10] Ito A, Ojima K, Naito H, Ichinose N, Tateishi T (2000). Preparation, solubility, and cytocompatibility of zinc-releasing calcium phosphate ceramics. J. Biomed. Mater. Res..

[11] Kuo MC, Yen SK (2002). The process of electrochemical deposited hydroxyapatite coatings on biomedical titanium at room temperature. Mater. Sci. Eng. C.

[12] Nie X, Leyland A, Matthews A (2000). Deposition of layered bioceramic hydroxyapatite/TiO_2_ coatings on titanium alloys using a hybrid technique of micro-arc oxidation and electrophoresis. Surf. Coat. Technol..

[13] Sobieszczyk S (2010). Surface modifications of Ti and its alloys. Adv. Mater. Sci..

[14] Rabiei A (2006). A study on functionally graded HA coatings processed using ion beam assisted deposition with in situ heat treatment. Surf. Coat. Technol..

[15] Bai X, More K, Rouleau CM, Rabiei A (2010). Functionally graded hydroxyapatite coatings doped with antibacterial components. Acta Biomater..

[16] Nan K (2009). Strontium doped hydroxyapatite film formed by micro-arc oxidation. Mater. Sci. Eng. C.

[17] Asmawi R, Ibrahim MHI, Amin AM, Mustafa N, Noranai Z (2017). Development of bioactive ceramic coating on titanium alloy substrate for biomedical application using dip coating method. IOP Conf. Ser. Mater. Sci. Eng..

[18] Askari N, Yousefpour M, Rajabi M (2017). Determination of optimum Al content in HA-Al_2_O_3_ nanocomposites coatings prepared by electrophoretic deposition on titanium substrate. Int. J. Appl. Ceram. Technol..

[19] Yoshimura M (2008). Formation of growing integrated layer (GIL) between ceramics and metallic materials for improved adhesion performance. Mater. Sci. Eng. B Solid State Mater. Adv. Technol..

[20] Sugiyama N, Yoshimura M (2009). Novel growing integration layer (GIL) method for joining/bonding of metallic and ceramic materials, and its applications for bulk metallic glasses with high bioactivities. Mater. Sci. Eng. B Solid State Mater. Adv. Technol..

[21] Huang C-H, Chen R-S, Yoshimura M (2017). Direct bioactive ceramics coating via reactive growing integration layer method on α-Ti-alloy. Mater. Sci. Eng. C.

[22] Tao ZS (2016). A comparative study of zinc, magnesium, strontium-incorporated hydroxyapatite-coated titanium implants for osseointegration of osteopenic rats. Mater. Sci. Eng. C.

[23] Yilmaz B, Evis Z, Tezcaner A, Banerjee S (2016). Surface characterization and biocompatibility of selenium-doped hydroxyapatite coating on titanium alloy. Int. J. Appl. Ceram. Technol..

[24] Bonifacio MA (2017). Gallium-modified chitosan/poly(acrylic acid) bilayer coatings for improved titanium implant performances. Carbohydr. Polym..

[25] Canalis E, Hott M, Deloffre P, Tsouderos Y, Marie PJ (1996). The divalent strontium salt S12911 enhances bone cell replication and bone formation in vitro. Bone.

[26] Xue W (2007). Preparation and cell-materials interactions of plasma sprayed strontium-containing hydroxyapatite coating. Surf. Coat. Technol..

[27] Ni GX, Lin JH, Chiu PKY, Li ZY, Lu WW (2010). Effect of strontium-containing hydroxyapatite bone cement on bone remodeling following hip replacement. J. Mater. Sci. Mater. Med..

[28] Prekajski M (2016). Ouzo effect-new simple nanoemulsion method for synthesis of strontium hydroxyapatite nanospheres. J. Eur. Ceram. Soc..

[29] Marie PJ, Garba MT, Hott M, Miravet L (1985). Effect of low doses of stable strontium on bone metabolism in rats. Min. Electrolyte Metab.

[30] Synhaeve N (2014). Chronic exposure to low concentrations of strontium 90 affects bone physiology but not the hematopoietic system in mice. J. Appl. Toxicol..

[31] Han Y, Hong SH, Xu K (2003). Structure and in vitro bioactivity of titania-based films by micro-arc oxidation. Surf. Coat. Technol..

[32] Ahu Akin F, Zreiqat H, Jordan S, Wijesundara MBJ, Hanley L (2001). Preparation and analysis of macroporous TiO_2_ films on Ti surfaces for bone-tissue implants. J. Biomed. Mater. Res..

[33] Grynpas MD, Marie PJ (1990). Effects of low doses of strontium on bone quality and quantity in rats. Bone.

[34] Han Y, Zhou J, Lu S, Zhang L (2013). Enhanced osteoblast functions of narrow interligand spaced Sr-HA nano-fibers/rods grown on microporous titania coatings. RSC Adv..

[35] Yan J, Sun JF, Chu PK, Han Y, Zhang YM (2013). Bone integration capability of a series of strontium-containing hydroxyapatite coatings formed by micro-arc oxidation. J. Biomed. Mater. Res. Part A.

[36] Yanovska A, Kuznetsov V, Stanislavov A, Danilchenko S, Sukhodub L (2012). Calcium-phosphate coatings obtained biomimetically on magnesium substrates under low magnetic field. Appl. Surf. Sci..

[37] Sun J, Han Y, Huang X (2007). Hydroxyapatite coatings prepared by micro-arc oxidation in Ca- and P-containing electrolyte. Surf. Coat. Technol..

[38] Zhao Q (2013). Preparation and properties of composite MAO/ECD coatings on magnesium alloy. Colloids Surf. B Biointerfaces.

[39] Huang Y, Yan Y, Pang X, Ding Q, Han S (2013). Bioactivity and corrosion properties of gelatin-containing and strontium-doped calcium phosphate composite coating. Appl. Surf. Sci..

[40] Kung KC, Lee TM, Chen JL, Lui TS (2010). Characteristics and biological responses of novel coatings containing strontium by micro-arc oxidation. Surf. Coat. Technol..

[41] Avci M, Yilmaz B, Tezcaner A, Evis Z (2017). Strontium doped hydroxyapatite biomimetic coatings on Ti_6_Al_4_V plates. Ceram. Int..

